# Demand analysis of public sports service for older adults using Kano model

**DOI:** 10.3389/fpubh.2025.1525447

**Published:** 2025-02-17

**Authors:** Xin Yang, Zimiao Leng, Changning Ma, Guanghui Yang

**Affiliations:** School of Physical Education, Yanshan University, Qinhuangdao, China

**Keywords:** demand analysis, public sports service, older adult sport, Kano model, global aging

## Abstract

**Aim:**

China is one of the countries with a relatively high degree of population aging in the world. Compared with other countries, China has the largest number of older adults and the fastest aging speed. In the increasingly serious situation of population aging, it has become the common desire of the whole society to actively develop sports for older adults and improve the health and quality of life of older adults. By identifying the needs of public sports services for older adults, this study ensures the sustainable improvement and meeting the needs of sports services for older adults in the future. The findings aim to improve the quality of life of older adults, enhance their physical and mental health.

**Methods:**

Participants were from Haigang District, Changli County, and Funing District of Qinhuangdao City, Hebei Province, China. A total of 436 older adults who met the survey requirements were included. The socio-demographic characteristics questionnaire was used to investigate and analyze the needs of the participants for public sports services.

**Results:**

Of the 23 quality attributes evaluated by participants, nine were “must quality,” two were “one-dimensional quality,” three were “attractive quality,” and nine were “indifferent quality.” The better values (satisfaction) ranged from 32.80 to 58.49%, and the worse values (dissatisfaction) ranged from 28.21 to 77.98%. In addition, the average ID of 23 service quality items was 2.25, and 12 attributes were above the average, which were high value-added attributes.

**Conclusion:**

Based on Kano model, it is found that older adults have a positive attitude toward physical activities, and the demand for different dimensions of public sports services is different. This study provides a theoretical basis for the decision makers of public sports services for older adults to formulate corresponding policies, and provides a scientific basis for sports instructors to optimize the content of public sports services and meet the personalized and characteristic needs of public sports services for older adults which contributes to healthy aging.

## Introduction

The World Health Organization notes that the pace of population aging is much faster than in the past, and that the global population of people aged 60 years and older will more than double from 900 million in 2015 to about 2 billion by 2050. An older adult is defined by the United Nations as a person who is over 60 years of age. PRC Law On Protection of the Rights and Interests of the Elderly stipulates that an older adult refers to citizen over age of 60. China is currently experiencing a significant demographic transition characterized by an increasingly aging population. By 2019, there were 254 million people aged 60 and above in China, and 176 million people aged 65 and above. By 2040, an estimated 402 million people in China, or 28% of the population, will be over 60 years old (1).

In the outline of The Healthy China 2030 Blueprint, it is clearly proposed to improve the physical fitness of the whole people and extensively carry out national fitness (2). The Chinese government has prioritized public sports services as a means to enhance national fitness and health. Since the introduction of the National Fitness Plan, various policies have aimed to integrate sports into daily life, encouraging public participation and improving health literacy among citizens. In this study, sports refers to physical activities carried out by older adults for the purpose of promoting physical health, delaying senility, preventing senile diseases and enriching leisure life. An appropriate recommendation for older adults includes moderately vigorous cardiorespiratory activities, strength and power training for maintenance of muscle mass and specific muscle-group performance, as well as balance-mobility practice and flexibility exercise as needed (3). Common forms of physical activities for older adults in China include square dancing, walking, chess and cards, gateball and so on. For instance, research indicates that national fitness policies have effectively increased public engagement and integrated fitness into community culture, with many provinces reporting heightened participation rates in sports activities from 2008 to 2017 (4).

The World Health Organization developed a definition of healthy aging. It is the process of developing and maintaining the functional ability that enables wellbeing in older age (5). Sports for older adults is a necessary measure to realize healthy aging (6). With the rapid development of social economy, sports has played an increasingly prominent role in promoting the advance of health (7), preventing and treating diseases (8), restoring physical and mental health (9), and extending life expectancy (10). More and more old people gradually have the idea of lifelong sports and sports life, and the demand for sports is more and more intense. Public sports service is a concept with Chinese characteristics. This study summarizes the definition of public sports service by Chinese scholars as products and behaviors provided to meet the needs of public sports. However, the imbalance between supply and demand and the unequal distribution of resources exist in sports public sports service of China, which leads to a large number of demands not being met. This requires us to recognize the current needs of older adults in sports services and provide accurate public sports services for older adults. Therefore, understanding the demand level of older adults for public sports services can not only help government agencies formulate more realistic sports service policies, provide sports services for older adults, and optimize the current level of public sports services, but also promote them to participate in sports activities (11), improve the health level and happiness of older adults, so as to improve the satisfaction of public sports services.

Based on this, this study proposes an analysis model based on the Kano model that is suitable for determining the content priority of public sports services for older adults in China. The main research objectives are as follows: (1) Taking the three districts of Qinhuangdao as an example, the Kano model was used to find out the public sports service needs that need to be improved and optimized based on the needs of older adults; (2) Explore the Kano model to provide reference suggestions for management decisions in the context of healthy aging; (3) Combining Kano model with public sports services to explore new application scenarios of Kano model in the field of sports can provide materials for subsequent researchers to comprehensively understand sports for older adults, provide method guidance for sports services for older adults, and promote the development of sports research for older adults in China.

## Literature review

### Public sports service

The aging population worldwide is growing rapidly, leading to increased demand for public services, including sports and recreation services tailored to older adults. Engagement in public sports services has been linked to substantial health benefits for older adults. A systematic review concluded that regular physical activity can reduce the risk of falls by approximately 23%, which is particularly critical for older adults who face heightened fall risks (12). The importance of physical activity in aging populations is well-documented, with numerous studies indicating that regular physical exercise significantly improves the physical and mental well-being of older adult individuals (13). The role of sports public services in promoting healthy aging is critical, as these services provide accessible and organized opportunities for physical activity, which is essential for maintaining health, preventing diseases, and improving the quality of life in older adults (14).

In China, the focus on healthy aging and public sports services for older adults has gained attention in recent years, with studies showing that well-organized sports programs can positively impact the health of older adults. Wei et al. found that improved accessibility and cleanliness of sports facilities significantly correlate with better mental health outcomes among community residents. Their study suggests that enhancing public sports services could lead to a reduction in mental distress, thus promoting overall well-being (15). Furthermore, Chen and Zhang reported that participation in community sports programs is associated with increased physical activity levels, contributing to better physical health and reduced incidence of lifestyle-related diseases (16). Wang and Wei highlighted that public sports services, such as fitness instruction and organized physical activities, play a crucial role in promoting physical activity among older adults. For many older adults, participating in physical activities is not just about maintaining physical health but also about staying socially engaged. Public sports services that promote social interaction and community involvement can enhance the mental well-being of older adult individuals and reduce feelings of loneliness and isolation (17).

In response to the advent of a rapidly aging society, older people are encouraged to participate in leisure activities, which can promote physical and mental health, improve quality of life, and contribute to health in later life (18). The results showed that men and women who frequently participated in sports groups (such as stretching calisthenics, gateball, and ground golf) and hobby clubs (including fancywork, ceramics, technical art, painting, and group singing) had significantly better physical health component summary scores (PCS) than women. Women had better mental health component summary scores (MCS) than those who did not participate (19). Older adults who walk three times a day for half an hour or even 10 min are healthier, less prone to illness, and stand out for their serenity and optimism (20). Physical activity is most effective for improving individual health in older adults (21). Lack of facilities was also found to be an important barrier to participation in physical activity and exercise. The Healthy China 2030 Blueprint sets the basic work target of having sports facilities of 2.6 square meters per capita by 2020, 2.2 square meters by 2025 and no less than 2.5 square meters by 2030 (22). Government departments play an important role in providing subsidies and funding for the development of free outdoor sports facilities. These facilities will provide more access for older adults to sports and promote health.

### Kano model

The Kano Model, developed by Professor Noriaki Kano in the 1980s, serves as a framework for understanding customer satisfaction and product development by categorizing customer preferences. This model identifies five types of categories: Must-Be Attributes, One-Dimensional Attributes, Attractive Attributes, Indifferent Attributes, and Reverse Attributes. These categories help in determining how different aspects of a service or product contribute to overall satisfaction. The model is particularly useful for product development and service design as it enables the prioritization of features based on their impact on customer satisfaction (23). Kano model divides product and service attributes into five categories:

Must-Be Attributes: These are the basic features that customers expect and are essential to customers (24). When must-be quality is provided, the satisfaction of older adults will not increase, while if it is not provided, the satisfaction of them with public sports services will be greatly reduced. Must-be quality is also the most basic need in the life of older adults. For instance, older adults may be dissatisfied if physical monitoring services are not provided in the community. However, it cannot be claimed that they will be satisfied when physical monitoring services are provided.

One-Dimensional Attributes: These attributes result in satisfaction when fulfilled and dissatisfaction when not met. The degree of customer satisfaction is directly proportional to the performance level of these attributes. One-dimensional qualities were what older adults thought they expected to receive in public sports services. When these qualities are satisfied, it will improve older adults’ satisfaction with public sports services. If not, it will cause their dissatisfaction with public sports services.

Attractive Attributes: These features delight customers when present but do not cause dissatisfaction if absent. They are often unexpected and can set a product or service apart from competitors. These attributes can significantly enhance the overall experience for older adult participants, as they go beyond basic expectations and offer added value.

Indifferent Attributes: Customers are neutral toward these attributes, and they neither contribute to satisfaction nor dissatisfaction.

Reverse Attributes: These are attributes that some customers may perceive negatively. Their presence can lead to dissatisfaction, while their absence may be seen positively by others.

**Figure 1:**
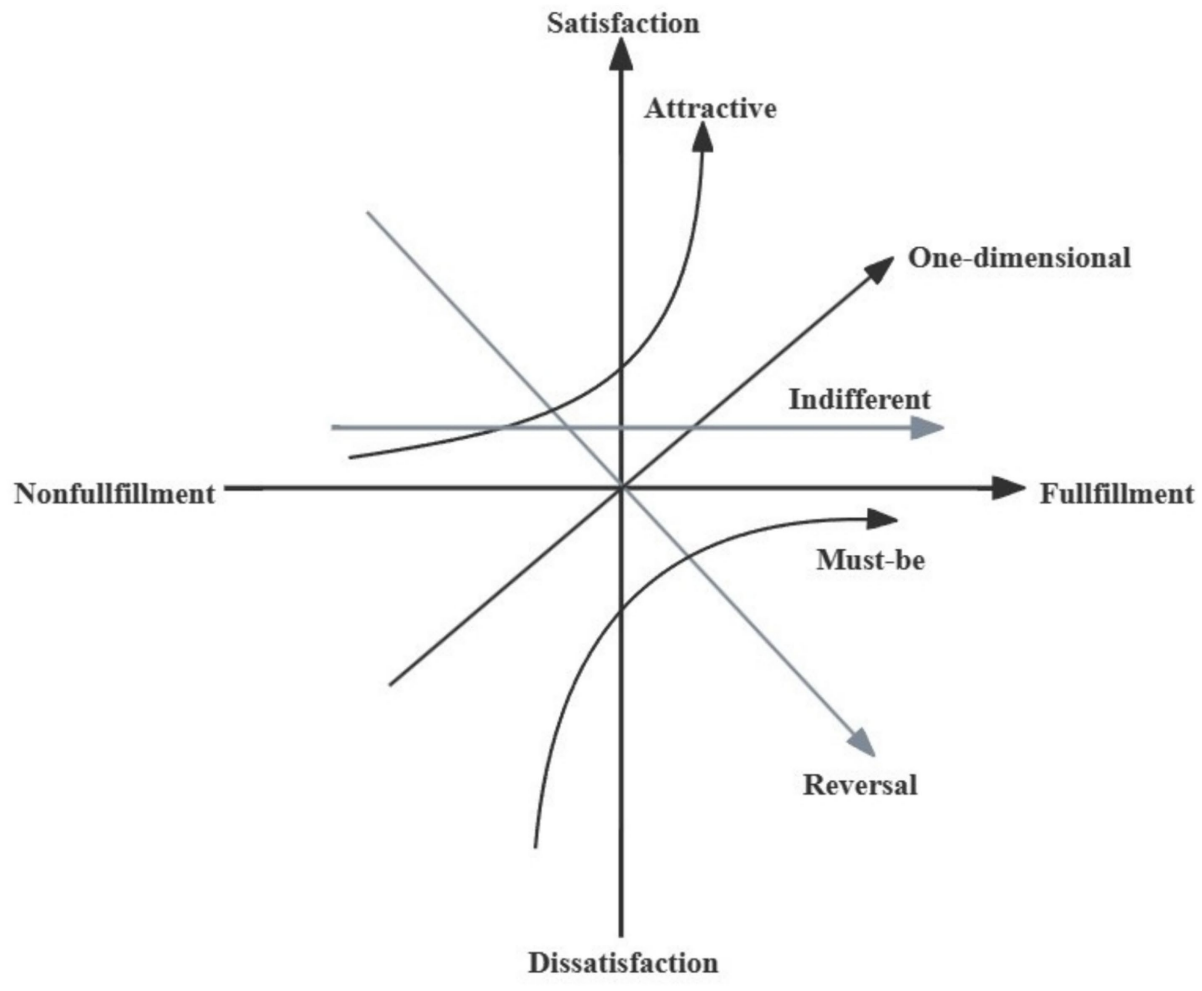
Kano model of quality attributes.

Kano model has a strong relevance to the satisfaction of older adults’ demand for public sports services. Kano model can help the government and relevant departments to clarify the quality attributes of public sports services, which makes the improvement of public sports services for older adults more strategic and targeted. The government or relevant departments should give priority to ensuring must-be quality, giving priority to one-dimensional quality, and providing attractive quality as far as possible (Figure 1).

The Kano model has been widely applied in different fields, including product development, service industries, healthcare, and education, to enhance customer satisfaction and align products and services with customer needs. One key strength of the Kano model is its focus on understanding latent customer needs, which are often not explicitly stated by customers but can significantly impact satisfaction (25). Kano model is especially relevant when addressing evolving consumer demands, particularly in sectors like sports services for older adults, where customer needs are diverse, and expectations shift based on health, mobility, and social engagement factors. In recent years, the model has been integrated with various methodologies, such as Quality Function Deployment (QFD), to further refine service or product offerings. For example, the combination of Kano model with QFD has been used to enhance the design of sports equipment, ensuring that user satisfaction is prioritized during development (26). The IPA model proposed by Martilla and James in 1977 is a comprehensive analysis method composed of two dimensions of Importance and Performance, which has been widely used in requirement decision analysis (27). The Kano model in combination with IPA analysis was used to explain customer rating behavior and identify priorities for attributes that needed improvement (28, 29). The model has also been adapted to different contexts, such as healthcare (30), e-service quality (31), mobile tourist guide application (32) et al., to better understand the factors influencing user satisfaction in those areas (33).

Its application in the sports sector has gained attraction, especially in enhancing customer satisfaction and experience. One notable study explores the integration of the Kano Model with Kansei Engineering and Taguchi methods to improve football spectator satisfaction. The research identifies emotional needs among spectators, categorizing them according to the Kano framework to enhance participation (34). The findings emphasize the emotional connection spectators have with the sport, which can inform strategies for engagement. Another application of the Kano Model categorizes the requirements of university students in sports programs, demonstrating its versatility beyond traditional customer satisfaction metrics. This study reveals how different aspects of program offerings can be aligned with student expectations, ultimately improving their satisfaction levels (35). Furthermore, the model has been used to analyze consumer demand for sports drinks, providing insights into quality attributes that affect consumer preferences. By applying the Kano Model, researchers highlighted the importance of safety, hydration, and natural ingredients in shaping consumer preferences for sports drinks in a competitive market (36). Recent research has also examined cultural differences in sports preferences through the lens of the Kano fuzzy model. This study proposes to adopt Kano fuzzy model to minimize the overriding effect of indifferent attributes, prevalent in a collectivist culture where respondents are reluctant to show their extreme attitude in neither positive nor negative (37). This adaptation of the model allows for a nuanced understanding of diverse consumer behaviors. In summary, the Kano Model has proven to be a valuable tool in the sports industry, facilitating deeper insights into customer preferences and guiding improvements in service delivery and product offerings. The ongoing exploration of its applications will likely yield further innovations in enhancing customer satisfaction in sports. By using Kano model, Yildiz et al. found that with the improvement of customers’ perception of the service quality of sports centers, their expectation level generally increased significantly. Customers expect the service they receive to be of high quality and to meet their expectations (38).

The Kano model can also be applied to evaluate and improve the public sports service system for older adults, particularly in terms of service delivery and management. As Liu and Wei demonstrated in their study, applying the Kano model to urban public sports services helps categorize service attributes based on their impact on user satisfaction (39). This approach enables service providers to focus on improving attributes that matter most to older adults, such as the frequency of sports activities, the professionalism of sports instructors, and the availability of social engagement opportunities. Applying the Kano model to public sports services provides a structured approach to understanding and addressing the diverse needs of older adults in public sports services. This model allows service providers to prioritize the development and enhancement of features that significantly impact satisfaction while ensuring that basic needs are adequately addressed. By using the Kano model to evaluate public sports services, service providers can better understand the diverse needs of older adults and prioritize the development of features that enhance satisfaction and participation. This approach not only improves the quality of services but also ensures that public sports services are inclusive and accessible to older adults, regardless of their physical or social limitations.

## Participants and methods

### Participants

Older adults in three different districts of Qinhuangdao City, Hebei Province, were selected in 2024. Participants met the following eligibility criteria: (1) age ≥ 60 years old (40); (2) being able to participate in physical exercise by themselves; (3) voluntary participation in the investigation. Subjects were excluded if they met the following exclusion criteria: (1) were in the acute phase of the disease; (2) those with communication, cognitive or mental disorders; (3) can not participate in physical exercise independently.

### Survey questionnaire

The questionnaire for this study was divided into two parts. The first part was a self-designed social demographic characteristics questionnaire, which included gender, age, education level, residence, living conditions, exercise duration, exercise frequency, etc. The second part is a survey on the demand for public sports services among older adults based on the Kano model, as shown in Table 1. It was designed based on the Kano model of Kano and a previous study by Chengwen et al. (41). Based on a more common understanding and from the perspective of the general government’s implementation of public management, sports public services can generally include the following six types of services: fitness facility services, fitness organization services, physical monitoring services, fitness guidance services, sports activities services, sports information service (42).

**Table 1:** Attributes in six dimensions.

Number	Attribute
A1	Surveillance sites into community services
A2	Provide physical fitness monitoring services
A3	Provide exercise prescription design
B1	Offer sports competitions
B2	Equipment conditions of the surrounding sites
C1	Provide physical activity information
C2	Physical activity information
C3	Sports travel information
C4	Provide health and fitness knowledge
C5	National and local sports policies and regulations
D1	Fitness Pathways
D2	Open space
D3	Fitness equipment
D4	The track and field
D5	Various courts (basket, foot, goalball, etc.)
E1	Motor skills training
E2	Explain sports rules
E3	Health education
E4	Scientific fitness guidance
F1	Official sports organizations
F2	Grassroots sports organizations
F3	Organize an exercise routine
F4	Organize and participate in sports activities (events)

The second part consists of 23 pairs of questions, each consisting of a forward question (functional form) and a reverse question (functional form), for example, “1.A. How would you feel if surveillance sites were provided into the community? 1.B. How would you feel if you did not?” For each question, respondents could choose the most appropriate answer from “I like that,” “It must be that,” “I am neutral,” “I can tolerate that,” and “I do not like that.” There are 25 possible outcomes, each corresponding to a Kano attribute. “M” represents must-be attribute, “O” represents the one-dimensional attribute, “A” represents the attractive attribute, “I” represents indifferent attribute, “R” represents the reverse attribute, and “Q” represents the suspicious answer, as shown in Table 2.

**Table 2:** Kano evaluation table.

Functional form	Dysfunctional form				
	I like it that way	It must be that way	I am neutral	I can live with it	I dislike it that way
I like it that way	Q	A	A	A	O
It must be that way	R	I	I	I	M
I am neutral	R	I	I	I	M
I can live with it	R	I	I	I	M
I dislike it that way	R	R	R	R	Q

In this study, Cronbach’s α coefficient was used to test the reliability, which was proposed by Cronbach (43) in 1951. The value interval is [0–1], and the closer it is to 1, the higher the reliability of the questionnaire is. The questionnaire data were imported into SPSS 26.0 for reliability test, and the Cronbach’s α coefficient values of the forward population and the reverse population of the demand for public sports services for older adults were calculated. The positive problem coefficients of each dimension ranged from 0.646 to 0.906, and the overall value was 0.906. The reverse question coefficients of each dimension ranged from 0.682 to 0.946, and the overall value was 0.958. The Cronbach’s α coefficient value greater than 0.6 indicated that the scale had internal consistency, between 0.7 and 0.8 indicated that the reliability was good, and between 0.8 and 0.9 indicated that the scale had very good reliability. In addition, the KMO (Kaier-Meyer-Olkin) value of kano questionnaire was 0.948, and the significance of Bartlett sphericality test was *p* < 0.001, indicating that the data were suitable for factor analysis. Therefore, the scale has good reliability, reliable test results, and internal consistency. After data collection, the Cronbach’s alpha of Kano questionnaire was calculated by SPSS software to verify the consistency of the scales used in the questionnaire. The Cronbach’s alpha values of the six dimensions were: A physical fitness monitoring service functional (0.837) and dysfunctional (0.682), B physical activity service: functional (0.646), dysfunctional (0.706), C sports information service: functional (0.785), dysfunctional (0.841), D sports facilities service: functional (0.712), dysfunctional (0.703), E sports guidance service: functional (0.804), dysfunctional (0.921), F sports organization service: functional (0.816), dysfunctional (0.946). The values represented by each dimension were considered acceptable (Table 3).

**Table 3:** Reliability and validity of the function and dysfunction of 6 dimensions of public sports services.

Dimension	Functional question	Dysfunctional question	Num
A. Physical monitoring services	0.837	0.682	3
B. Sports activities services	0.646	0.706	2
C. Sports Information Service	0.785	0.841	5
D. Fitness facility services	0.712	0.703	5
E. Fitness guidance services	0.804	0.921	4
F. Fitness organization services	0.816	0.946	4
Overall	0.906	0.958	23

### Statistical analysis

IBM SPSS Statistics 26.0 software was used to analyze the data. Frequencies and percentages were used to describe the sociodemographic characteristics of the participants. The Kano model was used to describe the attributes of public sports service needs of participants and to analyze the differences in the needs of public sports services for older adults.

### Methodology

The kano model explains how the relationship between objective performance and customer satisfaction with an attribute depends on customer evaluation of a product or service (44). In terms of data analysis, the traditional classification method of Kano et al. was first used to analyze the data, and then the satisfaction and dissatisfaction index proposed by Berger et al. (45) was used to analyze the data. Finally, the hierarchy of public sports service needs of older adults was sorted. It is important to note that due to the nature of non-probabilistic samples, the results of this study cannot be generalized.

### Investigation methods

Since most of older adults surveyed have a secondary school education or below, there is a serious psychological exclusion to the investigation. Therefore, the survey staff were systematically trained before the survey began, and the investigators were required to be very familiar with the content of the questionnaire. At the same time, the ability of local dialect is required in the selection of investigators in different regions. After homogenization training, researchers began to conduct the investigation. According to the GDP ranking of Qinhuangdao in 2023, the 9 districts of Qinhuangdao were divided into three parts, and an administrative district was randomly selected in each part. Haigang District, Changli County and Fanning District were selected as the survey objects. Questionnaires were distributed in parks, communities, activity centers and other places where older adults were concentrated in the three administrative districts. In the investigation process, the demand for public sports services of older adults was investigated in the form of one-to-one interviews or one-to-many lectures. The investigators would read the survey contents in the form of oral language, and the respondents would answer the questions. All the questionnaires were interviewed on the spot and collected on the spot. A total of 450 questionnaires were distributed from the three jurisdictions according to the administrative region, and 436 valid questionnaires were collected, with an effective questionnaire rate of 96.89%.

## Results

### The sociodemographic characteristics of the participants

A total of 436 older adults who met the requirements of this study completed the questionnaire. Among all participants, 52.29% were women. There were 253 older adults aged 60 to 65 years. One hundred and thirty-five participants were between 66 and 70 years of age; 11 participants were over 80 years of age. Among the participants, 297 (68.12%) had a secondary school education or below. There were 98 older adults with college degree or bachelor degree, accounting for 22.48%. The distribution of sociodemographic characteristics of the participants is shown in Table 4.

**Table 4:** Demographic characteristics.

Characteristics	Observation (N)	Percentage (%)
**Gender**		
Female	228	52.29%
Male	208	47.71%
**Age group (years)**		
60–65	253	58.03%
66–70	135	30.96%
71–75	23	5.28
76–80	14	3.21%
80 and above	11	2.52%
**Education level**		
Secondary school or below	297	68.12%
College or bachelor degree	98	22.48%
Master degree or higher	41	9.4%
**Current living condition**		
Living alone	48	11.01%
With spouse	237	54.36%
With children	125	28.67%
Other	26	5.96%
**Income**		
2,000 and below	75	17.20%
2,001–3,000	174	39.91%
3,001–4,500	100	22.94%
4,501–6,000	63	14.45%
6,001 and above	24	5.50%

### Analysis of the Kano model

As shown in Table 5, from the Kano final classification, we can identify nine public sports services classified as “must quality,” two as “one-dimensional quality,” three as “attractive quality” and nine as “indifferent quality.”

**Table 5:** Attribute classification of Kano indicators.

Dimension	Number	M	O	A	I	Category
Physical monitoring services	A1	153	114	74	95	M
	A2	118	152	71	95	O
	A3	105	87	134	110	A
Sports activities services	B1	142	100	82	112	M
	B2	96	73	74	193	I
Information consulting services	C1	131	100	86	119	M
	C2	132	95	95	114	M
	C3	78	68	82	208	I
	C4	63	61	96	216	I
	C5	78	73	69	216	I
Fitness facility services	D1	157	91	118	70	M
	D2	179	161	61	35	M
	D3	166	159	78	33	M
	D4	123	96	86	131	I
	D5	135	109	100	92	M
Sports activities services	E1	95	83	154	104	A
	E2	87	75	105	169	I
	E3	154	97	103	82	M
	E4	107	86	148	95	A
Fitness organization services	F1	70	110	101	155	I
	F2	83	122	78	153	I
	F3	101	150	105	80	O
	F4	113	99	78	146	I

The change of the importance degree (ID) value of Kano model can better reflect the importance of various public service needs, which is the main basis for the division of demand levels. Importance calculation can classify the attributes and levels of customer needs, so as to allocate public service resources reasonably and improve the accuracy of service and customer satisfaction.

When we determine that there is some correlation, we use numeric values, such as 5, 3, 1, and 0, to describe the relationship (46). Referring to the scholar’s calculation method of customer demand importance, this paper assigns values of 5, 3, 1, and 0 to the four demand types M, O, A, and I, respectively (47). The better value is the estimated ability of the feature to create satisfaction and calculated as: Equation 1. The worse value is the estimated value of the feature to create dissatisfaction if it is not included and calculated as: Equation 2 (48).


$$\mathrm{ID}\;\left(\mathrm{Importance Degree}\right)=\frac{F\left(M\right)*5+F\left(O\right)*3+F\left(A\right)*1+F\left(I\right)*0}{F\left(M\right)+F\left(O\right)+F\left(A\right)+F\left(I\right)}$$

(1) $$\mathrm{Better}=\frac{A+O}{A+M+O+I}$$

(2) $$\mathrm{Worse}=\frac{\left(-1\right)*\left(A+M\right)}{A+M+O+I}$$

**Figure 2:**
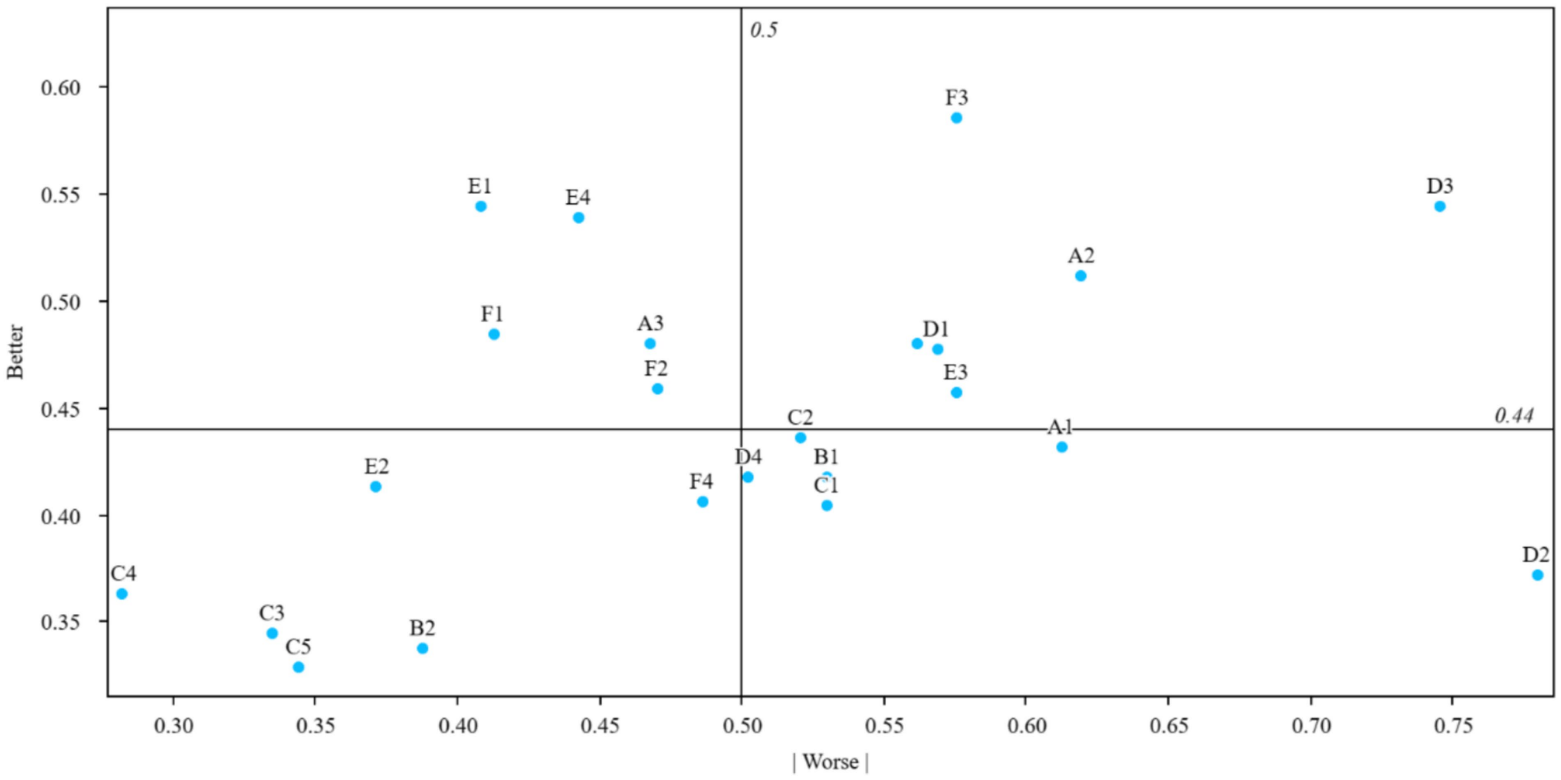
Attributes on the better-worse plot.

The intensity of classification was calculated according to Figure 2 distribution of types of public sports services. The total strength was calculated as the percentage of total responses in the three categories of “must-be,” “one-dimensional” and “attractive,” and ranged from 50 to 92%. Both better and worse values are between 0 and 1, with better values closer to 1 indicating greater satisfaction in providing the service, and worse values closer to 1 indicating that providing the attribute can only prevent dissatisfaction. A value close to 0 indicates that the service has little effect on satisfaction or dissatisfaction (Table 6).

**Table 6:** Attribute classification of Kano indicators and calculated values of Better and Worse for each quality.

Attribute	Better	Worse	Total satisfaction index	Category strength	ID
A1	43.12%	−61.24%	0.78	0.09	2.71
A2	51.15%	−61.93%	0.78	0.08	2.56
A3	47.94%	−46.79%	0.75	0.07	2.11
B1	41.74%	−52.98%	0.74	0.10	2.50
B2	33.72%	−38.76%	0.56	0.05	1.77
C1	40.37%	−52.98%	0.73	0.07	2.39
C2	43.58%	−52.06%	0.74	0.08	2.39
C3	34.40%	−33.49%	0.52	0.01	1.55
C4	36.24%	−28.21%	0.50	0.08	1.36
C5	32.80%	−34.40%	0.50	0.01	1.56
D1	47.71%	−56.88%	0.84	0.09	2.70
D2	37.16%	−77.98%	0.92	0.04	3.30
D3	54.36%	−74.54%	0.92	0.02	3.18
D4	41.74%	−50.23%	0.70	0.06	2.27
D5	47.94%	−56.19%	0.79	0.06	2.53
E1	54.36%	−40.83%	0.76	0.14	2.01
E2	41.28%	−37.16%	0.61	0.04	1.75
E3	45.64%	−57.57%	0.81	0.12	2.67
E4	53.90%	−44.27%	0.78	0.09	2.16
F1	48.39%	−41.28%	0.64	0.02	1.79
F2	45.87%	−47.02%	0.65	0.09	1.97
F3	58.49%	−57.57%	0.82	0.10	2.43
F4	40.60%	−48.62%	0.67	0.03	2.16

## Discussion

### One-dimensional qualities

The satisfaction of the participants is proportional to the “One-dimensional quality,” that is, the more “one-dimensional quality” provided, the higher the satisfaction of the participants. Conversely, dissatisfaction increases when provision is inadequate (48). In this study, “physical fitness monitoring station” and “organizing daily exercise” were “one-dimensional qualities.” Compared with other public sports services, these two programs put more emphasis on the social emphasis on health management and active lifestyles of older adults. Older adults have a higher demand for physical activities guidance, which may be due to the lack of the number of social sports instructors. As an organizer, instructor and disseminator of social sports, social sports instructors are an important type of talents in sports undertakings in China (49). Chinese President Xi Jinping stressed that effectively dealing with the aging population can not only improve the life and quality of older adults, but also promote economic development and enhance social harmony. Physical fitness monitoring service can not only promote older adults to actively participate in sports, but also lay a foundation for the development of sports for older adults in China, improve the physical health monitoring mechanism.

### Must-be qualities

“Must-be quality” is the basic feature of a service or product. Participants perceived them as a given or a must have service. In this study, “monitoring points into community services,” “interesting competition activities,” “surrounding venues and equipment,” “sports activity information,” “fitness path,” “open space,” “fitness equipment,” “various courses,” “fitness knowledge teaching” are “essential qualities.” The results of national physical fitness surveillance were the essential elements. National physical fitness surveillance is the work of the state to systematically grasp the national physical condition by sampling survey and according to the national physical fitness monitoring indicators promulgated by the state, to conduct systematic testing on the monitored objects and analyze the monitoring data regularly throughout the country. Physical fitness monitoring belongs to preventive active behavior, which can prevent diseases and improve physical function by formulating exercise prescriptions, carrying out targeted and effective exercise and diet improvement.

### Attractive qualities

“Attractive quality” means that participants would be very surprised and satisfied if these qualities were provided. If they fall short, the user will not be dissatisfied. In this study, “exercise prescription design,” “exercise skills training,” “scientific fitness instruction” were attractive features compared to other public sports services. Scientific fitness guidance plays a vital role in the participation of older adults in sports activities. Scientific fitness guidance is an important prerequisite for older adults to carry out sports activities. Strengthening the personnel training construction of social sports instructors can effectively guide and promote older adults to participate in sports activities. In addition, regular exercise skills training can improve the enthusiasm of older adults to participate in sports activities, promote emotional communication between older adults, help to reduce the loneliness of older adults, better bring older adults together, help older adults integrate into family life, and improve life happiness. The findings suggest that the current number of social sports instructors in the community or subdistrict does not meet the needs of older adults. Secondly, older adults put forward the need for exercise prescription design and hoped to increase the prescription design of related exercise.

### Indifferent qualities

“Indifferent quality” refers to a service that the user does not care much about whether it is provided or not. Customers are neutral toward these attributes, and they neither contribute to satisfaction nor dissatisfaction. Therefore, indifferent quality is not the priority to be satisfied in public sports services. “Competitive sports,” “sports tourism information,” “provision of health and fitness knowledge,” “national and local sports policies and regulations,” “track and field,” “explanation of sports rules,” “official sports organizations,” “grassroots sports organizations,” “organization of participation in sports activities” are indifferent qualities. The absolute values of the Better coefficient and the Worse coefficient of these nine services were lower than the average level of the six dimensions. The provision or non-provision of the service did not affect older adult satisfaction. According to the life cycle of Kano type, I → A → O → M, indifferent quality can be transformed to attractive quality to a certain extent. Demand will change with the time, and time will also affect the functional demand, and the Kano type of service content will also change. Therefore, after the development of the other three qualities, the government or relevant departments can focus on the unrelated quality project dimensions, provide better sports services for older adults, pay attention to the changes in the needs of older adults, and improve their satisfaction.

### Attributes on the better-worse plot

All services were divided into four quadrants, with nine aspects classified as “Must-be qualities,” two as “One-dimensional qualities,” and three as “Attractive qualities,” Nine were classified as “Indifferent qualities.” None of the aspects were classified as reverse qualities. Quality of service attributes have their own life cycle and change over time. The attractive quality theory predicts that the role of different quality attributes in the product life cycle will change (50). Each service attribute has its own life cycle and changes accordingly over time (51). There may be some qualities that are attractive at first, but over time, eventually become essential. This means that the quality attribute of public sports services for older adults is not static. We need to understand the dynamic development of the public sports needs of older adults, to explore all kinds of difficulties faced by older adults in public sports services, and to provide them with public sports services more suitable for older adults, more conducive to older adults’ satisfaction with public sports services and their health.

## Suggestions

First of all, sports facilities are important conditions and guarantees for older adults to participate in physical activities. Sports departments should include suitable sports equipment for older adults (such as walkers, upper limb tractors, sit-to-stand back massagers, etc.) in fitness circle programs. Government departments should rationally plan sports facilities in each district according to the number, economic status, distribution and fitness habits of older adults in each district. According to the health status of older adults, it is equipped with sports equipment that is easy to operate and safe. At the same time, the government departments should integrate the site resources and provide older adults with equipment and venues for recreational activities, which can not only improve the utilization rate of the site, but also meet the needs of older adults for sports space. In addition, the government departments should make efforts to create 15-min fitness circles, which helps motivate older people to participate in physical activities.

Secondly, with the development of social economy, older adults are paying more attention to their health status and regularly monitor their physical fitness. Based on the physical fitness monitoring report, older adults can conduct health assessment and make exercise prescriptions, which will promote the participation in physical activities. With the aggravation of population aging, physical fitness surveillance station has become an important part of community health management. In November 2023, the home care service center in Haigang District of Qinhuangdao, Hebei Province, was put into use. The service center contains intelligent equipment such as electronic physical examination machines and traditional Chinese medicine physical examination machines (52). Physical fitness monitoring services provide health assessment and feedback for older adults, which helps to enhance their health awareness. Regular health examination and feedback can encourage older adults to actively participate in daily health management and improve self-care ability.

Finally, social sports instructors play an important role in older adult sports. They organize physical activities to arouse the enthusiasm for older adults. At the same time, they also popularize scientific sports knowledge and provide correct and standardized sports guidance for older adults to avoid injuries. Sports departments should train social sports instructors and constantly strengthen their professionalism and standardization. Colleges should add sports related courses suitable for older adults and promote the professional development of social sports instructors. The community should increase the number of social sports instructors to teach older adults sports knowledge. In addition, sports departments should strengthen assessment of social sports instructors and carry out regular training. This helps social sports instructors to provide better sports services for older adults.

## Conclusion

Based on Kano model, it is found that older adults has a positive attitude toward physical exercise, and the demand for different dimensions of public sports services is different. This study provides a theoretical basis for the decision makers of public sports services for older adults to formulate corresponding policies, and provides a scientific basis for sports instructors to optimize the content of public sports services and meet the personalized and characteristic needs of public sports services for older adults which contributes to healthy aging.

## Data Availability

The raw data supporting the conclusions of this article will be made available by the authors, without undue reservation.
